# TFIP11 Interacts with mDEAH9, an RNA Helicase Involved in Spliceosome Disassembly

**DOI:** 10.3390/ijms9112105

**Published:** 2008-11-04

**Authors:** Xin Wen, Sissada Tannukit, Michael L. Paine

**Affiliations:** University of Southern California School of Dentistry, Center for Craniofacial Molecular Biology, 2250 Alcazar Street, CSA room 103, Los Angeles, California 90033-1004, USA. E-Mails: xwen@usc.edu (X. W.); tannukit@usc.edu (S. T.)

**Keywords:** mDEAH9, Dhx15, G-patch, Ntr1, pre-mRNA splicing, Prp43, spliceosome disassembly, Spp382 and TFIP11

## Abstract

Yeast proteins Ntr1, Ntr2 and Prp43 function in spliceosome disassembly. An Ntr1-Ntr2 protein complex recruits Prp43 to allow the removal of the lariat-intron in late-stage RNA splicing activity. Based on amino-acid sequence similarities across species, TFIP11 and mDEAH9/Dhx15 have been identified as homologues of yeast Ntr1 and Prp43, respectively. The *N*-terminal region of TFIP11 contains a G-patch, which is a highly conserved domain of many RNA-processing proteins. TFIP11 displays a unique and characteristic subnuclear localization pattern, in close proximity to SC35 nuclear speckles. Transfected GFP-tagged mDEAH9 displays an evenly distributed nuclear localization and is excluded from the nucleoli; however when TFIP11 and mDEAH9 are co-transfected, both proteins colocalize to distinct nuclear speckles. These data show that TFIP11 recruits mDEAH9 suggesting that these two proteins have similar biological activities to their yeast counterparts.

## 1. Introduction

In a number of proteomic studies, human TFIP11 (hTFIP11) has been identified as a component of the spliceosome [1, 2]. The yeast homologue of TFIP11 is Ntr1/Spp382 (encoded by the *YLR424W* gene in *S. Cerevisiae*) and shares about 30% amino acid similarity with mammalian TFIP11 proteins. The *N*-terminal region of TFIP11 contains a G-patch, which is a highly conserved domain of many RNA-processing proteins. The *C. elegans* homologue to TFIP11 is referred to as STIP (septin and tuftelin interacting proteins) [3]. STIP has a speckled nuclear localization pattern similar to TFIP11 [3, 4]. RNA interference studies targeting STIP in *C. elegans* show morphological abnormalities starting at about the 16-cell stage and 100% embryonic lethality [3]. This lethal phenotype could be faithfully rescued using either a *Drosophila* or human TFIP11 coding sequence under the control of the native *C. elegans* STIP promoter. These data provide direct evidence that STIP/TFIP11 has a highly conserved nuclear function across animal species from worms and flies, to humans. In yeast, Ntr1 has been shown to interact directly with Prp43, an ATP-dependent RNA helicase [5–8]. This interaction results in the recruiting of Prp43 to the spliceosome, and is a required step for the release of the lariatintron and spliceosome disassembly [9]. Mouse DEAH9 (a protein produced from gene locus *Dhx15*) is the mouse homologue of Prp43, and mDEAH9 restores normal function of Prp43 in a mutant, yeast-based splicing assay [10]. Using a GFP-tagged TFIP11 protein, we previously have shown that TFIP11 locates in novel subnuclear speckles that are distinct from, but in close proximity to SC35 speckles [4]. In addition, based on an *in vivo* splicing assay [11], TFIP11 functions as a splicing factor [4].

We report here that the localization of exogenously added TFIP11-fluorescent fusion protein is spatially representative of endogenous TFIP11 expression. TFIP11 deletion studies have identified amino acid regions essential for the characteristic punctated nuclear distribution of TFIP11. Mutations to the highly conserved G-patch domain of TFIP11 have no impact on this distinctive nuclear localization. Like their yeast counterparts, TFIP11 interacts with mDEAH9 *in vitro,* and colocalizes with mDEAH9 *in vivo.*

## 2. Experimental Section

### 2.1. Plasmid Constructs

The mouse TFIP11-C1 vector has been previously described [4]. A TFIP11 G-patch mutation plasmid was engineered using QuickChange site-directed mutagenesis kit (Stratagene, La Jolla, CA) using the plasmid TFIP11-C1 [4] as template DNA. The primers used to create this construct (designated mTFIPmut forward and reverse) are listed in Table 1.

The mouse TFIP11-DsRed construct was cloned by removing the TFIP11 cDNA from the TFIP11-N1 vector [4], using Eco RI and Age I restriction enzymes, and subcloning this cDNA into pDsRed-N1 plasmid (Clontech) using the same restriction sites of the multicloning site.

A CMV-driven mouse TFIP11 cDNA construct, with 3 repeats of the FLAG epitope at its C-terminus, was prepared for coimmunoprecipitation studies. The TFIP11 cDNA, covering the entire open reading frame, was released from the construct TFIP11-N1 [4], and subcloned into the FLAG-containing expression vector pCMV-3Tag-8 (Stratagene) using appropriate restriction enzyme sites. The resulting construct is referred to as TFIP-FLAG.

A EGFP-tagged, and Myc-tagged full-length mDEAH9 vectors were prepared by RT-PCR using total RNA extracted from LS8 cells [4], and using primers listed in Table 1 to cover the entire reading frame of mDEAH9 (GenBank accession # AF017153). Forward primers included the restriction site Eco RI. Both PCR-generated mDEAH9 cDNAs were first cloned into pCR2.1-TOPO vector (Invitrogen), and subsequently subcloned into the vector pEGFP-C1 (Clontech), and the vector pCMV-Myc (Clontech) at Eco RI/Kpn I multicloning site; and these constructs are referred to as mDEAH9-C1 and Myc-DEAH respectively.

All plasmid construct cDNA inserts were sub-cloned by standard methodologies [12], and sequenced in their entirety to ensure no nucleotide errors were introduced and the inserts were in the correct reading-frame.

### 2.2. Immunoprecipitation and Western Blot

Transfected cells were lysed with RIPA buffer (10 mM Tris-HCl pH 7.6, 150 mM NaCl, 1% NP-40, 1% sodium deoxycholate, 0.1% SDS) in the presence of multiple protease inhibitors (Santa Cruz Biotechnology Inc., Santa Cruz, CA), and DNA was sheared by repeated aspirations through a 21-gauge needle. The cell suspension was spun at 10,000 g for 5 minutes, and the supernatant collected. In the case of the FLAG immunoprecipitation (IP) assay, the supernatant was mixed with anti-FLAG M2 antibody (Sigma-Aldrich, St. Louis, MO) for 1 hour at 4 °C, followed by the addition of protein A/G agarose beads and the continuous agitation for an additional 1 hour at 4 °C. In the case of IP with Myc antibody, agarose conjugated anti-Myc (Santa Cruz Biotechnology, Inc., Santa Cruz, CA) was used. The immunoprecipitate was then pelleted at 10,000g for 5 minutes, and then washed in RIPA buffer for four times in the presence of protease inhibitors. The final pellet was resuspended in 2x Laemmli sample buffer with 2-mercaptoethanol and boiled for 3 minutes before loading to SDS-PAGE gel. Collected proteins samples were transferred to Immobilon-P transfer membrane (Millipore, Billerica, MA) using Hoefer TE77 semi-dry transfer unit and Western analysis was performed using ECL system (Amersham Biosciences, Piscataway, NJ).

### 2.3. Transfection, Immunocytochemistry and Imaging

HEK293 cells were transfected using Lipofectamine 2000 as previously described [4], and then incubated for 24 hours prior to imaging. Immunocytochemistry was performed using the Histostain kit (Zymed Laboratories, Invitrogen Corporation, Carlsbad, CA). Prior to imaging, cells were fixed with 4% paraformaldehyde, washed with phosphate buffered saline (PBS), mounted in VECTASHIELD medium (Vector Labs, Burlingame, CA), and sealed with nail varnish. Confocal images were captured as previously described [4].

## 3. Results

### 3.1. The localization of the transfected TFIP11-EGFP fusion protein mimics that of endogenous TFIP11

**Figure 1.**
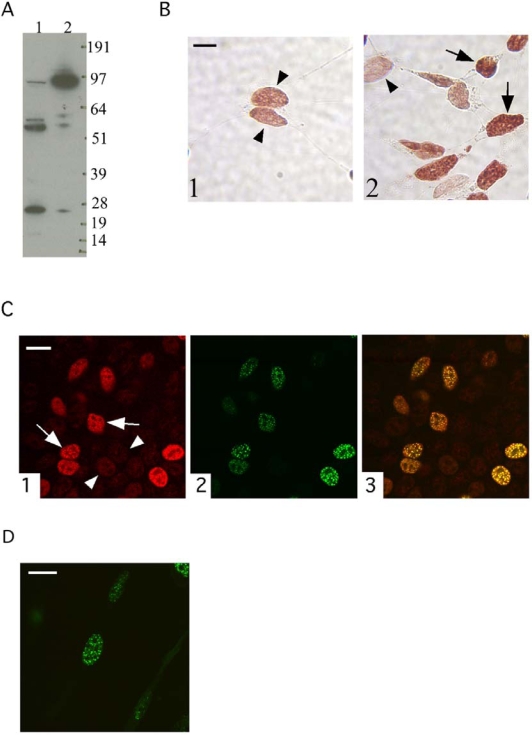
Immunoreactivity, and subcellular localization of TFIP11. (A) Western blot analysis, and (B) immunocytochemistry using the TFIP11 antibody. (C) Confocal microscopic images of TFIP11-C1, and (D) G-patch mutant TFIP11-C1 transfected cells. Scale bar: panel B 10 μm; panels C and D 20 μm.

Previously we showed that TFIP11-GFP fusion proteins localized to unique speckles domains in the cell nucleus [4]. Here, a rabbit generated anti-peptide antibody was generated against amino acid residues 32–45 [LQNEFNPNRQRHWQ] of mouse TFIP11 (Zymed Laboratories Inc, South San Fransisco, CA). This peptide domain shares 100% identity with TFIP11 from multiple species, including rat and human. The resulting anti-peptide polyclonal antibody was used to detect both endogenous and a transfected CMV-driven human TFIP11 cDNA construct (purchased from Origene, Rockville, MD; catalogue # SC112654) in HEK293 cells by Western blot analysis (Figure 1A, lanes 1 and 2 respectively). The band corresponding to full-length TFIP11 is seen at ~ 96–97 kDa. Three smaller bands were also seen in the cell lysate of both preparations (Figure 1A). Note that the loading of transfected cell lysate was one tenth of the untransfected cell lysate in order to visualize full-length TFIP11 clearly in both samples.

Immunocytochemistry using the TFIP11 anti-peptide antibody against non-transfected (Figure 1, panel B1) and TFIP11-C1 transfected (Figure 1, panel B2) HEK293 cells indicated that the endogenous TFIP11 localized in the cell nucleus in a speckled pattern. Non-transfected (arrow-heads) and transfected (arrows) cell nuclei are indicated (Figure 1, panel B). As can be seen in Figure 1, panel B2, an identical pattern of immunoreactivity was observed in the nucleus of non-transfected and transfected cells with a stronger signal being seen in the transfected cells (arrows) compared to non-transfected cells (arrow heads). This TFIP11 nuclear expression pattern was further confirmed by confocal microscopy. TFIP11-C1 transfected HEK293 cells were observed using confocal microscopy using the TFIP11 primary antibody and Alexa fluro 610-conjugated secondary antibody (seen as red; Figure 1, panel C1), and direct fluorescence (seen as green; Figure 1, panel C2). The merged image is shown (Figure 1, panel C3). Non-transfected (arrow-heads) and transfected (arrows) are indicated (Figure 1, panel C1). This data indicates that the spatial localization of TFIP11-EGFP is representative of endogenous TFIP11 localization.

### 3.2. The G-patch of TFIP11 is not essential for the characteristic nuclear distribution of TFIP11

A mutational study targeting the highly conserved G-patch domain of TFIP11 was performed to determine if this domain was responsible for the characteristic speckled nuclear distribution of TFIP11. Using the TFIP11-C1 vector as template, three glycine residues within the G-patch domain, G166, G168, and G170, were mutated to alanine, alanine, and arginine, respectively in a single construct. Confocal microscopy was used to observe the cellular localization G-patch mutant TFIP11-C1 vector transfected into HEK293 cells with direct fluorescence (Figure 1, panel D). This G-patch mutant demonstrated the identical nuclear localization speckled pattern to the wild type TFIP11-C1 [4], suggesting that the G-patch is not responsible for the cellular localization TFIP11 (Figure 1, panel D).

### 3.3. TFIP11 colocalizes with mDEAH9 in vivo, and interacts with mDEAH9 in vitro

**Figure 2.**
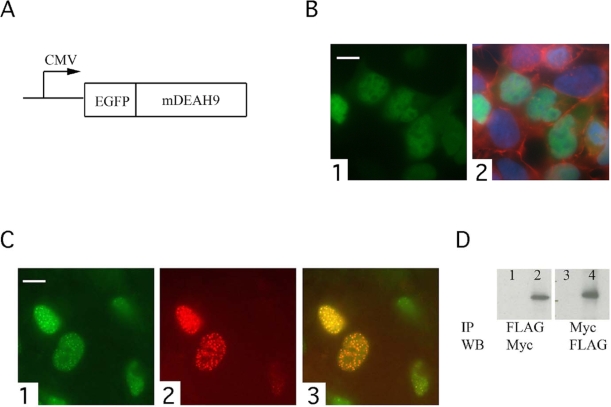
TFIP11-mDEAH9 interactions. (A) Schematic representation of mDEAH9-C1 construct. (B) Subcellular localization of transfected mDEAH9. (C) mDEAH9-C1 and mouse TFIP11-DsRed cotransfected cells. Scale bar, panels B and C 10μm. (D) HEK293 cells cotransfected with TFIP-FLAG and Myc-DEAH followed by IP and WB.

The yeast homologue of TFIP11, Ntr1/Spp382, has been shown to recruit Prp43 to spliceosome. The helicase activity of Prp43 is required for spliceosome disassembly and lariatintron release [7]. The mouse homologue of Prp43 is mDEAH9, which is able to restore functionality in Prp43 mutant yeast [10]. To examine whether TFIP11 interacts with mDEAH9, analogous to the previously identified yeast Ntr1-Prp43 interaction, an mDEAH9-EGFP fusion construct (mDEAH9-C1; Figure 2A) was used to determine the cellular localization of mDEAH9 . HEK293 cells were transfected with mDEAH9-C1, counterstained with DAPI and phalloidin, and observed under fluorescence microscope (Figure 2, panel B). Green signal indicated mDEAH9-C1 localization (Figure 2, panels B1 and B2), blue identifies DAPI-stained nuclei, and red identifies phalloidin-marked actin in the cytoplasm (Figure 2, panel B2). In figure 2, panels B 1 and B2 are the same cell population. HEK293 cells were also cotransfected with mDEAH9-C1 (green fluorescence; Figure 2, panel C1) and mouse TFIP11-DsRed (red fluorescence; Figure 2, panel C2). The merged image is shown (Figure 2, panel C3). Unlike TFIP11, which localizes to distinct nuclear speckles, transfected mDEAH9 has an evenly distributed nuclear localization and is absent from the nucleoli (Figure 2, panel B). However, when TFIP11 and mDEAH9 are overexpressed following cotransfection, both introduced proteins colocalize to the characteristic TFIP11 speckled nuclear domains (Figure 2, panel C3).

Coimmunoprecipitation experiments were performed using HEK293 cells cotransfected with FLAG-tagged (TFIP-FLAG) and Myc-tagged mDEAH9 (Myc-DEAH) constructs, followed by immunoprecipitation (IP) and Western blot (WB) using antibodies as indicated (Figure 2, panel D lanes 2 and 4). Controls were HEK293 cells cotransfected with either pCMV-3Tag-8 and Myc-DEAH (Figure 2, panel D lane 1), or pCMV-Myc and TFIP-FLAG (Figure 2, panel D lane 3). Using either an antibody against the FLAG or Myc epitopes, a direct protein-protein interaction between TFIP11 and mDEAH9 could be demonstrated (Figure 2, panel D lanes 2 and 4).

These results are supportive of TFIP11 having a functional activity similar to its yeast homologue (Ntr1) in being able to recruit mDEAH9 to the late-stage spliceosome, and initiate events including the release of the lariatintron and spliceosome disassembly.

## 4. Discussion

In this study we have relied heavily on identifying a biological function for TFIP11 and mDEAH9/Dhx15 by comparing these two proteins to their yeast homologues, Ntr1 and Prp43 recpectively. Notably, Ntr1 and TFIP11 contain a G-patch that is a feature of many RNA-processing proteins [13]. Here we demonstrate that the characteristic speckled nuclear location of TFIP11 is independent of the G-patch. Other likely functions of the G-patch include mediating protein-protein interactions with other splicing components. In the case of TFIP11 and mDEAH9, the G-patch may be directly responsible for recruiting mDEAH9 to theTFIP11-associated, late-stage spliceosome complex. This is a testable hypothesis. This is also not unprecedented, as this function has been demonstrated for the G-patch domain of yeast Spp2 directly interacting with Prp2 [14]. Prp2 is an RNA helicase protein related in sequence and function to Prp43. SPP2 and Prp2 function together in the first transesterification reaction of pre-mRNA splicing [14].

## 5. Conclusions

We conclude from published data using the yeast splicosome as a model system [5–10, 14], and colocalization and coimmunoprecipitation data presented here, that TFIP11 and mDEAH9 likely function together in late-stage RNA splicing events. TFIP11 appears to be a resident of the spliceosome complex, and is capable of recruiting mDEAH9 when required. Assuming the physiological functions of mammalian TFIP11 and mDEAH9 are similar to their yeast homologues (Ntr1 and Prp43 respectively) [5–8], it is likely that once mDEAH9 is recruited to the spliceosome, this then allows for the release of the lariatintron and subsequent spliceosome disassembly.

## Figures and Tables

**Table 1.** Synthetic oligonucleotides for PCR with restriction sites underlined.

mTFIPmut forward	5′- CTACGTCCCTGCGCGTGCCCTGCGGAAGAACGCAC
mTFIPmut reverse	5′- GTGCGTTCTTCCGCAGGGCACGCGCAGGGACGTAG
EGFP_mDEAH9 forward	5′- GAATTCTATGTCCAAGAGGCATCGGTT
Myc_mDEAH9 forward	5′- GAATTCTTATGTCCAAGAGGCATCGGTT
EGFP/Myc_mDEAH9 reverse	5′- TCACCAACATTCTGGCTTGATA
